# HIV vaccination: Navigating the path to a transformative breakthrough—A review of current evidence

**DOI:** 10.1002/hsr2.70089

**Published:** 2024-09-23

**Authors:** Godfred Yawson Scott, Dominic Worku

**Affiliations:** ^1^ Department of Medical Diagnostics Kwame Nkrumah University of Science and Technology Kumasi Ghana; ^2^ Infectious Diseases Department Morriston Hospital, Heol Maes Eglwys Morriston United Kingdom; ^3^ Public Health Wales Cardiff United Kingdom

**Keywords:** AIDS, HIV, immune system, infections, vaccines

## Abstract

**Background and Aim:**

Human immunodeficiency virus (HIV) remains a significant global health challenge, with approximately 39 million people living with HIV worldwide as of 2022. Despite progress in antiretroviral therapy, achieving the UNAIDS “95‐95‐95” target to end the HIV epidemic by 2025 faces challenges, particularly in sub‐Saharan Africa. The pursuit of an HIV vaccine is crucial, offering durable immunity and the potential to end the epidemic. Challenges in vaccine development include the lack of known immune correlates, suitable animal models, and HIV's high mutation rate. This study aims to explore the current state of HIV vaccine development, focusing on the challenges and innovative approaches being investigated.

**Methods:**

In writing this review, we conducted a search of medical databases such as PubMed, ResearchGate, Web of Science, Google Scholar, and Scopus. The exploration of messenger ribonucleic acid vaccines, which have proven successful in the SARS‐CoV‐2 pandemic, presents a promising avenue for HIV vaccine development. Understanding HIV‐1's ability to infiltrate various bodily compartments, establish reservoirs, and manipulate immune responses is critical. Robust cytotoxic T lymphocytes and broadly neutralizing antibodies are identified as key components, though their production faces challenges. Innovative approaches, including computational learning and advanced drug delivery systems, are being investigated to effectively activate the immune system.

**Results and Conclusions:**

Discrepancies between animal models and human responses have hindered the progress of vaccine development. Despite these challenges, ongoing research is focused on overcoming these obstacles through advanced methodologies and technologies. Addressing the challenges in HIV vaccine development is paramount to realizing an effective HIV‐1 vaccine and achieving the goal of ending the epidemic. The integration of innovative approaches and a deeper understanding of HIV‐1's mechanisms are essential steps toward this transformative breakthrough.

## 1 INTRODUCTION

Human immunodeficiency virus (HIV) stands as one of the most significant medical challenges of the 20th century, prompting extensive research and progress unmatched by many other illnesses, aside from the recent SARS‐CoV‐2 pandemic. Since its initial identification in the 1980s, HIV has been the subject of thorough investigation due to its unique viral life cycle, which is characterized by reverse transcription and integration of linear HIV DNA into the host DNA. This key mechanism provides a latent reservoir of virus, which is resistant to elimination and makes HIV incurable with a profound impact on both the innate and adaptive immune systems, culminating in symptom development. This virus not only leads to a gradual depletion of immune defenses but also triggers maladaptive immune responses. Consequently, individuals infected with HIV progress towards acquired immunodeficiency syndrome (AIDS), characterized by heightened susceptibility to a broad spectrum of infectious and noninfectious complications. These complications, which encompass bacterial, fungal, viral, and parasitic infections, can affect virtually any organ system, with a particular predilection for the central nervous system (CNS) and hematological manifestations.

At the end of 2022, an estimated 39 million people were living worldwide with HIV, with 1.3 million new cases diagnosed and 630,000 deaths.^1^, ^2^ When we consider HIV epidemiology, while globally represented, it remains despite progress concentrated within sub‐Saharan Africa (SSA). Here despite advances and declines in cases, great heterogeneity exists. In 2018 HIV incidence and mortality ranged from 2.8 to 1585.9/100,000 people and 0.8 to 676.5/100,000 people, respectively, highlighting the need for renewed approaches for HIV control.^3^ In 2014, the United Nations Programme on HIV/AIDS (UNAIDS) 2020 “90‐90‐90” goal was established whereby 90% of people with HIV were to be diagnosed, 90% of these should be on therapy of which 90% would have an undetectable viral load. While ambitious, progress was slow, with only 60 of 170 countries having reached this target by 2018, with heterogeneity noted in achieving this amongst different members of a given population with suboptimal improvements in HIV awareness, HIV incidence, and AIDS‐related deaths noted. Indeed, global access to effective generic antiretroviral therapy (ART) remains suboptimal and requires active progress.^4^, ^5^, ^6^, ^7^

The transition from the 90‐90‐90 goals to the 2025 95‐95‐95 targets reflects a significant shift in the global response to HIV/AIDS, particularly emphasizing the need to address geospatial inequalities and enhance healthcare access for marginalized populations.^8^ The UNAIDS report from July 2023 indicates that achieving the 95‐95‐95 targets globally remains a challenge, with progress being uneven across different regions and populations, especially among high‐risk groups such as sex workers.^8^, ^9^ As of 2021, Botswana, Eswatini, Rwanda, and Tanzania have all reached the 95‐95‐95 target, with several other African states on target to achieving this key target, which is important given the burden of HIV in Africa.^10^ Therefore, it is evident that there is a need for the development of robust infrastructure, adequate funding, and innovative healthcare design to facilitate the screening and engagement of at‐risk populations. This involves dismantling existing barriers and enhancing access to timely treatment, both at the population level and for individuals.

Since the pivotal Strategic timing of the antiretroviral treatment trial, the timing of therapy has drastically altered and is now advocated at the time of diagnosis irrespective of baseline CD4 count and may even be provided the same day, minimizing potential harms and immune dysfunction from delaying treatment until quantitative immune cell deficits have been detected providing a near normal life expectancy.^11^ Initial ART therapy at present is typically tripartite in nature, with the World Health Organization (WHO) advocating in their 2021 recommendation for a nucleoside reverse transcriptase inhibitor backbone in addition to Dolutegravir an integrase inhibitor with dual regime therapy possible in patients who are stable on therapy providing contraindications do not exist.^12^ This daily administration of several active agents helps to target different aspects of the HIV life cycle, thereby reducing the risks of emerging resistance. However, while ART is efficacious and has reduced HIV total mortality, it is known to result in important metabolic complications, including hyperglycemia, hypertriglyceridemia, and weight gain, which further increase the cardiovascular disease incidence (e.g., acute myocardial infection and ischemic stroke) already seen in HIV positive individuals. While the underlying mechanism of this raised cardiovascular risk is debated, it is increasing and is particularly evident in distinct subpopulations, namely female gender those who are older and have a pre‐existing raised body mass index, with the British HIV Association highlighting the need for annual cardiovascular risk assessment utilizing risk assessment tools (e.g., QRISK2) score, HbA1c assessment and lipid profile in all patients over 40 to allow for risk stratification and utilization of adjuvant therapies, e.g., statins where required.^13^, ^14^, ^15^ In addition, the need for high compliance to daily therapy is challenging and provides patients with a daily reminder of their lifelong condition. At present, 95% adherence is required to achieve sustained viral suppression, with data suggesting that 45% of people have poor adherence to therapy, with risk factors for this including low socioeconomic status, racial/ethnic minorities, and prior criminal justice involvement.^16^, ^17^, ^18^ The importance of good compliance with therapy is implicit in both to minimize the risks of resistance and ongoing transmission.

It is given these issues that new modes of therapy have been designed, such as injectable therapies, which hope to sustain suppressed viral loads while reducing associated pill burden. At present bimonthly Cabotegravir plus Rilpivirine is the only marketed injectable therapy, although several novel agents, including lenacapavir a capsid inhibitor, are under development. Importantly, long‐term data shows that these offer comparable efficacy and safety data in treatment naïve and experienced patients to traditional ART and may even be offered as a pre‐exposure prophylaxis agent.^19^, ^20^, ^21^ However, there are some issues, notably the need for healthcare involvement in the provision of the injections, lack of data regarding use in special populations (e.g., pregnancy), and the possibility of leading to resistance if improperly utilized.^22^ Therefore, while injectable therapies are an important part of the HIV armamentarium likely allowing for improved uptake of services by traditionally difficult‐to‐reach groups, the need for new approaches to HIV treatment is required.

Vaccination outside of improvements in hygiene and sanitation is the most successful approach to disease prevention and control.^23^ It is through vaccination that disease eradication, elimination, and control have been achieved. The ability, therefore, to provide an HIV vaccine is abundantly advantageous, as it could allow for durable, efficacious sterilizing immunity and thus protection on challenge and reduce the emergence of drug resistance and onward transmission risk. Therefore, if correctly designed and utilized, a vaccine could function complementary and additively to existing methods of control (e.g., Pre‐Exposure Prophylaxis), allowing for the possibility of ending the HIV epidemic while allowing for considerable economic savings. It would also remedy some of the shortcomings of existing methods (e.g., uptake of male circumcision and treatment as prevention). This has been the dream of the last 40 years, with two immunization strategies considered, namely a prophylactic vaccine and a therapeutic vaccination, with the latter allowing for improved disease control in patients with poor adherence and existing immune dysfunction or rebound viremia. The challenges, however, in vaccination development are notable chief among which are the lack of known correlates of immune protection in HIV, the lack of suitable sustainable animal models, and the high mutation rate of HIV.^2^

Indeed, the ability of HIV to evade the adaptive immune response and establish latency is of great importance to designing a vaccine and achieving viral control.^24^ Given the associated costs of vaccine development, the deployment of robust and likely efficacious vaccination designs must be utilized. For instance, it is clear that compared to conventional vaccine types (e.g., inactivated and live attenuated vaccines), messenger ribonucleic acid (mRNA) vaccines offer huge benefits being scalable in production, easy to design, stable in vivo, and can lead to durable T and B cell responses while also stimulating the innate immune system.^25^ This allows for the simultaneous targeting of HIV antigens and the possible induction of broadly neutralizing antibodies (bnAbs), which may augment vaccine efficacy.^26^ Indeed, mRNA vaccination during the recent SARS‐CoV‐2 pandemic has been fundamental in altering the disease phenotype, saving lives, and ending the pandemic.^25^ Therefore, any HIV vaccine must have several characteristics including activity against multiple HIV strains, ability to induce humoral and cytotoxic cellular responses to both block HIV‐1 infection and kill infected cells and thus combat the extreme genetic diversity of HIV, augment mucosal immunity where the majority of transmission and viral replication takes place, be safe, durable and cost‐effective. While this is challenging it has been estimated that in South Africa, a biannual vaccine with moderate efficacy (70% vaccine efficacy) achieving 20% population coverage could avert ~500,000 HIV cases over 10 years.^27^ HIV vaccination, despite recent advances, offers a sustainable solution to the HIV pandemic. This study aims to explore the current state of HIV vaccine development, focusing on the challenges and innovative approaches being investigated.

## 2 NATURAL LIFE CYCLE OF HIV AND CELL TROPISM

The HIV‐1 life cycle can be broadly classified into two main stages: the pre‐integration phase, which includes events from the HIV‐1‐mediated CD4 receptor and CXCR4 or CCR5 coreceptor interaction and signaling to the integration of the HIV‐1 genome into the host cell which includes,^28^, ^29^, ^30^ and the late stages of the virus life cycle, which consider HIV‐1 genome replication until viral egress from the cell and its maturation thereafter.^31^, ^32^, ^33^ Cell tropism in HIV‐1 is influenced by the usage of co‐receptors, with CXCR4 primarily facilitating infection in CD4+ T cells, while CCR5 is mainly associated with macrophage infection. Notably, during the course of natural infection, there is a shift in co‐receptor usage; early in the infection, CCR5 is predominantly utilized, which is linked to a higher risk of transmission from mucosal sites. As the infection progresses, CXCR4 usage becomes more prevalent, correlating with a more rapid decline in CD4+ T‐cell counts. Additionally, in a subset of elite controllers, the presence of the CCR5 delta 32 deletion significantly reduces viral kinetics, showcasing its impact on disease progression.^34^, ^35^

The pre‐integration and post‐integration stages can be further classified into 11 phases which include binding/attachment, fusion, trafficking, nuclear import, reverse transcription, integration, transcription/translation, assembly, budding, and release.^36^

**Figure 1:**
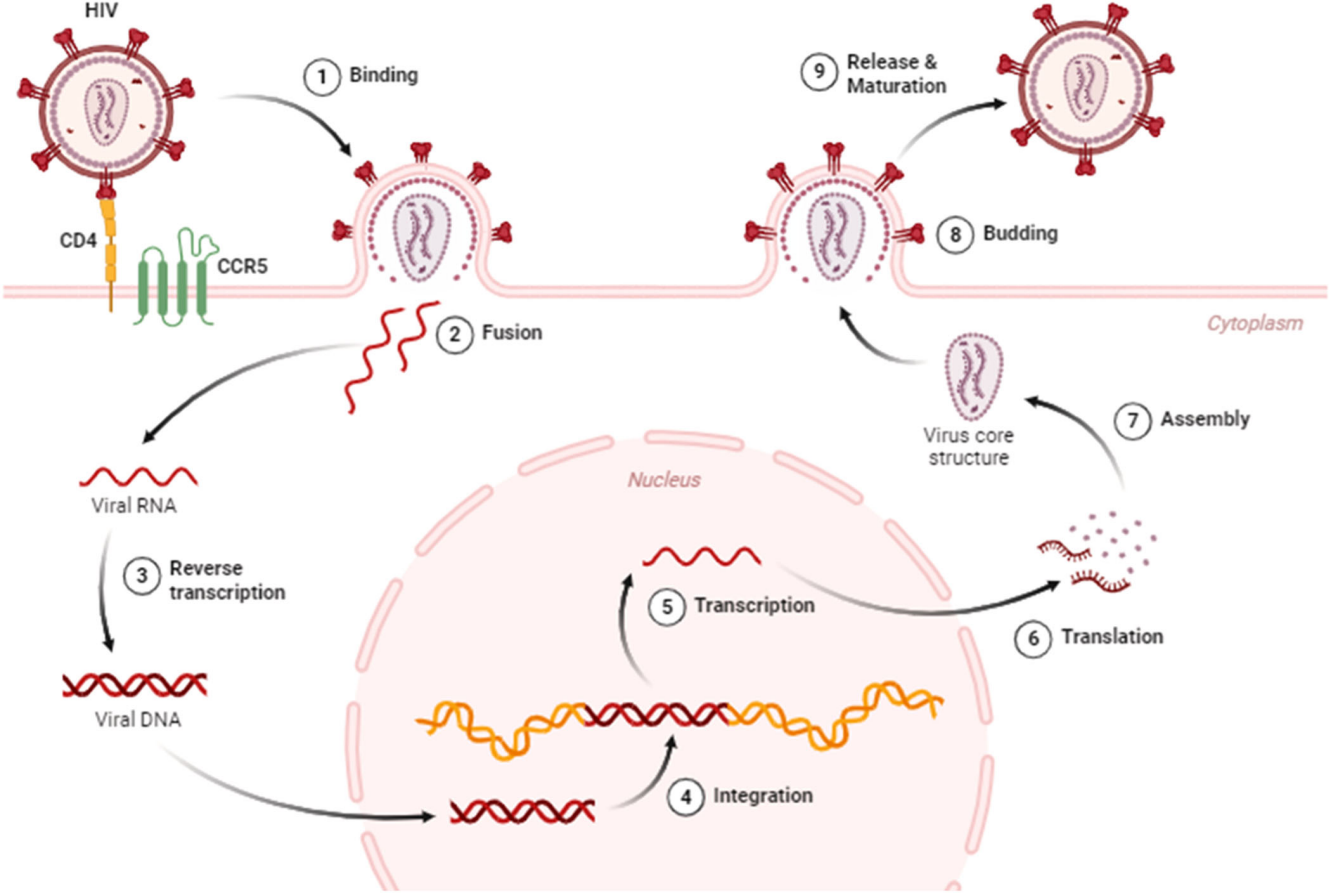
The HIV‐1 life cycle. The virus interacts with the host cell receptors (1), causing the virus to fuse and release its viral core into the host cell's cytoplasm (2). The core is then transported across the cytoplasm as reverse transcription and nuclear import begin (3). The viral components enter the nucleus through the nuclear pore and are localized (4) to transcriptionally active chromatin while uncoating and reverse transcription occur (5). The viral genes are then transcribed and translated (6) into the Gag polyproteins, which assemble (7) and localize to the host membrane, followed by the budding of an immature virion (8). During the final phase of the HIV‐1 lifecycle, known as maturation, the viral protease cleaves the Gag polyprotein into its component, functional proteins (9). The figure was originally designed with Biorender.

The virus attaching itself to the co‐receptor and CD4+ T cell receptor is the first stage of HIV infection, as shown in Figure 1. After interacting with the CD4 receptor on the surface of T cells, the viral envelope glycoprotein, or gp120, undergoes a conformational change that enables it to bind to either the CXCR4 or CCR5 co‐receptor.^37^ When the gp41 subunit is exposed as a result of this binding, the viral and cellular membranes fuse.^37^, ^38^ The viral genome and its enzymatic components, including reverse transcriptase (RT), integrase (IN), and protease (PR), are released into the cytoplasm of the host cell and moved to the nucleus once the viral envelope and host cell membrane fuse. After the RT transforms the viral RNA genome into double‐stranded DNA (dsDNA), the pre‐integration complex, a nucleoprotein complex made up of both host and viral proteins, and the viral genome facilitates the integrase enzyme integrate the dsDNA into the host cell genome.^39^, ^40^, ^41^ A provirus, which is the integrated viral DNA, is dormant until the host cell activates it.^42^, ^43^ mRNA, which is subsequently spliced and transported out of the nucleus into the cytoplasm, is created by the transcription of the provirus by the host cell RNA polymerase II enzyme.^43^ The host cell's ribosomes translate the viral mRNA into viral proteins. The structural proteins, such as the matrix (MA), capsid (CA), and nucleocapsid (NC), are created when the viral protease enzyme cleaves the Gag polyprotein. Reverse transcriptase, integrase, and protease are among the viral enzymes that are produced through cleavage of the Gag‐Pol polyprotein.^44^ At the host cell membrane, the viral RNA genome and proteins are assembled to form virions. The nucleocapsid core is formed when the viral proteins, such as Gag and Gag‐Pol, attach to the viral RNA genome.^45^, ^46^, ^47^, ^48^ The glycoproteins that make up the viral envelope are contained in a lipid bilayer that envelops the core and is derived from the host cell membrane. At last, the fully developed virions develop an envelope and emerge from the host cell membrane.^45^, ^46^, ^47^, ^48^

## 3 IMMUNE MODULATION AND IMMUNE CHANGES UPON HIV INFECTION

The main reason HIV hasn't had a cure for decades is its capacity to elude the immune system.^49^, ^50^ HIV contains several in‐built strategies for evading or disabling the host immune system.^51^, ^52^ HIV infection is characterized by a declining immune system, which can result in cancer, autoimmune disorders, and opportunistic infections. HIV‐I infects, reduces in number, and functionally compromises a subset of T lymphocytes derived from the thymus that express the CD4 cell‐surface molecule. Important recognition and induction tasks are carried out by CD4 T cells in the immune response to foreign stimuli.^53^, ^54^ HIV infection causes progressively declining CD4 T cell counts and immunological dysresponsiveness that eventually paralyzes almost every immune system component. This results in an increased risk of developing opportunistic infections and through reductions in immune surveillance and control of oncogenic viruses (e.g., Human papillomavirus, Epstein Barr‐Virus, and Human Herpesvirus 8), the development of cancers such as lymphoma, cervical cancer, and Kaposi sarcoma, which themselves are indicator conditions for HIV testing.^13^, ^55^ The clinical spectrum of HIV infection includes both asymptomatic infection and severe immunodeficiency, along with the infectious and malignant complications that are specific to AIDS.^56^, ^57^, ^58^, ^59^ A corresponding degree of CD4 T cell depletion reflects this range of clinical severity. Therefore, from an immunologic standpoint, HIV‐l immunodeficiency is seen as a graded severity of CD4 T cell depletion that is distributed over time, influenced by unknown cofactors, and linked to a spectrum of increasing clinical severity that corresponds to the degree of CD4 T cell depletion.^59^ While rare “elite controllers” who make up 0.2%–0.5% of HIV‐I patients demonstrate the ability to sustain viral load suppression and CD4+ counts in the absence of ART for many years. While this has been hypothesized to be due to defective HIV virus replication and host‐related factors, elite controllers demonstrate the possibility of HIV‐1 cure through their broad, specific, and superior T‐cell, innate cell, and natural killer cell effector functions, which a vaccine should hope to replicate. Despite this reprieve, elite controllers will eventually progress and lose virological control, likely through the deleterious effects of chronic immune activation and immune exhaustion. Indeed elite controllers are at increased risk of all causes of hospitalization versus ART recipients.^60^, ^61^, ^62^

HIV can subvert the host immune system by suppressing the expression of major histocompatibility class (MHC) class I and II molecules, proteins vital to immune cell recognition, and antigen presentation by antigen‐presenting cells, e.g., dendritic cells, Langerhans cells, and macrophages.^63^, ^64^, ^65^ At the molecular level, this is accomplished by several mechanisms, one of which is HIV's capacity to obstruct MHC class I and II gene transcription and translation, thereby lowering the total expression of these molecules on the surface of viruses.^66^ The HIV accessory protein Nef,^67^ which is crucial for viral pathogenesis due to its multiple interactions with cellular machinery and is thus a possible target for antiretroviral drug development^68^ MHC I and II molecules, CD3, CD4, and CD8 receptors are redirected by Nef to the endocytic pathway for degradation by Nef's interaction with their cytoplasmic tails. While Nef:Adaptor Protein‐2 interactions lead to tetherin and CD28 being sequestered and downregulated, respectively, all of which culminate in enhanced HIV‐1 replication, reduced T cell activation, and enhanced HIV virion budding.^69^, ^70^, ^71^, ^72^ HIV‐1 Nef‐mediated downregulation of cell surface MHC‐I molecules has been explained in part by the combined action of Nef and Phosphofurin Acidic Cluster Sorting Protein 1, which usurps the ADP ribosylation factor 6 endocytic pathway through a process that is dependent on phosphatidylinositol‐3 kinase and downregulates cell surface MHC‐I to the trans‐Golgi network.^73^

Additionally, the HIV Vpu protein promotes MHC class I molecule degradation by interacting with the host protein beta‐TrCP, which attracts the E3 ubiquitin ligase complex to tag MHC I for degradation in the proteasome. In addition, Vpu sequesters MHC‐I intracellularly in the early stages of endocytosis and recycling obstructing the movement of freshly synthesized MHCI molecules to the cell surface, where they are necessary for recognition by immune cells.^74^, ^75^ Targeting the host protein tetherin (also known as BST‐2 or CD317), Vpu obstructs the movement of freshly synthesized MHC I molecules from the endoplasmic reticulum (ER) to the cell surface.^76^ The membrane protein tetherin prevents virus particles from escaping from infected cells, thereby stopping the spread of the virus. By attaching to tetherin and encouraging its breakdown via the proteasome pathway, Vpu negates this antiviral mechanism.^77^ This makes it more difficult for cytotoxic T cells (CTLs) to present viral antigens, which aids the virus's ability to elude immune surveillance.^78^ The ubiquitin‐proteasome pathway is a complex interplay of three ubiquitin enzymes that function to add multiple ubiquitin molecules to suitable substrates (e.g., damaged proteins) and, in doing so earmark them for proteasome for breakdown as without which they would compromise cellular activities, e.g., cell division, antigen presentation. In HIV‐1, the Proteasome is exploited with all stages of HIV‐1 infection ultimately affecting cellular proteasome function by altering the balance of host and viral proteins. Moreover, all HIV‐encoded proteins interact with and attenuate proteasome function, which they may do so directly or indirectly. For instance, HIV‐1 transactivator of transcription (TAT) protein can increase IL‐7R degradation, decreasing infected CD8 T‐cell survival while enhancing viral replication; additionally, Vif can induce degradation of APOBEC3G, which is a cellular restriction factor and otherwise will be incorporated into new virions. Furthermore, there is evidence of UPS being used to establish HIV‐1 latency.^79^, ^80^

The combination of lymphocyte apoptosis and impaired lymphopoiesis in the thymus and bone marrow can significantly hinder the recovery of CD4 T cells. Concurrently, the immunoevasive strategies employed by HIV‐1 create a challenging environment for ongoing viral replication, complicating efforts to design effective vaccines. Developing a vaccine that can not only restore immune function but also target latent reservoirs established early in infection is essential for achieving sustained and optimal therapeutic efficacy against HIV‐1.^81^, ^82^

## 4 PROBLEMS WITH THE HIV RESERVOIR

One of the biggest obstacles to finding a treatment or maintaining long‐term viral control is the HIV reservoir. ART effectively suppresses viral replication in the bloodstream; however, the virus retains its reservoir in some cells (e.g., immune cells) tissues (e.g., gut‐associated lymphoid tissue) and lymph nodes and sanctuary sites (e.g., CNS), which may persist even despite effective ART.^83^ The definition of a viral reservoir is one where replication of a competent virus continues outside that of the main pool of the virus.^84^ These reservoirs continue to be a latent source of the virus, mostly found in long‐lived immune cells such as memory CD4+ T cells. The primary obstacle is that these reservoirs continue to exist despite intensive treatment, making total eradication impossible. Because of the reservoirs' long lifespan, a more thorough comprehension of the cellular and molecular processes at play is required for the creation of focused therapeutic interventions.^85^ Recent work has identified specific phenotypic signatures in infected memory CD4 T cells in ART‐experienced individuals with upregulation of surface CD44 and IL‐21 receptors. As such these may represent possible targets for designed therapeutics in the future.^86^

The initial indication of the formidable challenge of completely eradicating HIV from infected individuals surfaced in 1997. Three independent studies revealed the persistence of a latent and inducible viral reservoir in all participants, despite effective ART maintaining undetectable plasma viremia for several years.^87^, ^88^, ^89^ Subsequent longitudinal research affirmed the prolonged existence of the latent HIV reservoir in individuals with clinically effective ART, dampening hopes for virus eradication through antiretroviral drugs alone.^90^ It is for this reason that the production of therapeutic HIV‐I vaccines to control the viral reservoir is highly sought after. While the precise origin of re‐emerging plasma viremia remains unclear, it is widely accepted that latently infected, quiescent CD4+ T cells and other viral reservoirs play a role in the swift viral rebound typically observed within 2 weeks after stopping ART.^91^ Various mechanisms contribute to the persistence of HIV reservoirs during ART, encompassing the intrinsic stability of latently infected, quiescent CD4+ T cells, periodic homeostatic proliferation of reservoir‐sustaining cells, replenishment of infected cells via residual viral replication, insufficient penetration of antiretroviral drugs into viral replication privileged sites in tissues, and the persistence of HIV in sanctuary sites across different tissue compartments.^90^, ^92^, ^93^, ^94^, ^95^

The inability to eradicate or reduce the size of HIV reservoirs through latency‐reversing agents (LRAs) implies that reactivating latent HIV does not effectively stimulate the activation of CTLs to eliminate infected cells. While enhancing CTL responses against HIV to eliminate cells induced to express the virus with LRAs is a compelling idea, there are significant challenges.^96^ First, in cases where ART is initiated late after infection, CTL escapes mutants tend to dominate the latent viral reservoir. Secondly, there is a growing understanding that HIV may exploit immune‐privileged sites like B cell follicles in lymph nodes for persistence. Studies with nonhuman primates infected with simian immunodeficiency virus (SIV) have shown that B cell follicles are inaccessible to virus‐specific CD8+ T cells, and continuous viral replication occurs in CD4+ follicular helper T cells of elite controller animals with effective SIV‐specific CD8+ T cells. Hence, while it is crucial to induce broad CTL responses, there must also be a strategy to temporarily overcome essential immune regulatory mechanisms designed to prevent immunopathology in sites crucial for developing adaptive immune responses. Lastly, a notable complication arises with certain LRAs, particularly histone deacetylase inhibitors such as romidepsin and panobinostat. While these agents can reactivate segments of the latent HIV reservoir, they do not enhance cytotoxic T lymphocyte (CTL) activity, which further limits their effectiveness. This lack of increased CTL response poses a challenge in effectively targeting and eliminating reactivated HIV‐infected cells.^96^ What remains unclear, however, is the forces that assist in shaping the reservoir in both ART naïve and experienced patients and their contribution to multi‐drug resistance, reactivation, and degradation with evidence that HIV‐I decay is not associated with immune activation and may persist for decades.^97^

## 5 GENETIC DIVERSITY AND HOW IT CIRCUMVENTS NATURAL IMMUNE RESPONSES

HIV‐1 is diverse and exists in four groups (M, N, O, and P) and nine clades (A–H and J–K), of which clades C and A are the most prevalent globally and in particular in Southern Africa/India and East Africa/Russia respectively. The genetic disparity between such clades can be 25%–35% with further variability introduced through recombination allowing for the emergence of common/unique recombinant forms which often co‐exist in a given geographical area and are enriched in key populations who may be exposed to different HIV‐1 subtypes (e.g., people who inject drugs).^98^, ^99^ This is important as HIV‐1 subtypes can be associated with altered viral behavior, drug resistance (e.g., clade C and evafirenz resistance), and thus ART response (more rapid virological suppression with Clade C vs. A/B), and disease progression (e.g., clade D faster disease progression and dementia development vs. clade A).^100^

The variability in HIV‐1 is primarily attributed to the highly error‐prone nature of its reverse transcriptase enzyme. This enzyme is crucial for viral replication as it converts single‐stranded genomic RNA into double‐stranded viral DNA, subsequently integrated into the host genome.^101^ Notably, inhibitors of HIV‐1 reverse transcriptase play a vital role in ART. HIV‐1 reverse transcriptase is a multifunctional enzyme with RNA‐dependent and DNA‐dependent DNA polymerase activities, along with RNase H activity responsible for degrading the RNA strand of RNA/DNA hybrids.^101^ Unlike other DNA polymerases, HIV‐1 reverse transcriptase lacks proofreading ability, contributing to its error‐prone nature. The absence of proofreading, combined with the high rate of virus production during infection, leads to the continuous generation of new viral variants.^102^ The rate of nucleotide substitutions induced by reverse transcriptase is approximately 10^−4^ per nucleotide per replication cycle, resulting in one nucleotide substitution per genome per cycle. Additionally, insertions, deletions, and duplications contribute to the genetic diversity of HIV‐1.^103^ HIV‐1 exhibits rapid turnover, generating about 10^9^ virions per day in an infected individual. The short lifespan of plasma virus and virus‐producing cells (approximately 2 days) leads to almost complete replacement of wild‐type strains by drug‐resistant variants within 2–4 weeks.^102^ During antiretroviral treatment, the rapid turnover of the virus combined with its high mutation rate significantly contributes to the emergence of HIV variants that are resistant to these drugs. Throughout the infection, the production of viral quasi‐species has varying effects; in the early stages, mutations can aid in evading the immune response, but at the cost of viral replication efficiency. In individuals who develop resistance to ART, a relatively low level of virus is observed upon withdrawal of treatment, indicating a reduced viral fitness and replicative capacity, which offers some protective advantage to the host. However, as time progresses and host immunosuppression increases, compensatory mutations within HIV‐1 occur, enhancing viral fitness by improving pathogenicity, restoring replication capabilities, and accelerating disease progression.^104^, ^105^

Genetic recombination emerges as another potent force shaping HIV diversity, particularly in the context of drug resistance.^106^ Within each retroviral particle, there are two copies of single‐stranded RNA, and during reverse transcription, frequent template switches occur. This process results in mutations and recombination through both intramolecular and intermolecular jumps. Recombination can connect drug‐resistant mutations in HIV‐1, enhancing resistance to specific drugs. Moreover, it may give rise to multidrug‐resistant variants. Additionally, recombination can lead to the acquisition of mutations that compensate for a decline in viral fitness or replicative capacity caused by previously acquired resistance mutations.^106^

In its continuous evolutionary struggle, HIV employs sophisticated tactics to avoid detection by the immune system. The virus accumulates escape mutations in critical regions, particularly in the viral envelope glycoprotein gp120. This strategic mutation enables HIV to escape detection and neutralization by antibodies. The genetic diversity inherent in HIV facilitates antigenic variation, allowing the virus to alter its surface antigens and outsmart the host's immune system.^107^ This constant adaptation ensures the persistent infection of host cells and, significantly, enables the virus to evade immune memory. The identification of suitable vaccine targets is therefore key with any proposed targets required to be both antigenic and conserved amongst HIV strains. The use of artificial intelligence and predictive modeling can help tremendously in vaccine design by estimating antigenicity, immune responsiveness, construct stability, and associated toxicity. This could drastically reduce the time to vaccine development and has been trialed in Rhizopus, staphylococcal argenteus, and klebsiella pneumonia.^108^

## 6 IDENTIFICATION OF SUITABLE IMMUNE CORRELATES OF PROTECTION

The development of effective strategies against infectious diseases, particularly complex viruses like HIV, relies heavily on identifying immune correlates of protection and precise vaccine targets. In the dynamic province of HIV, characterized by significant genetic diversity and intricate immune evasion mechanisms, the quest for appropriate targets and correlates becomes increasingly complex.

Viral vaccine vectors, such as adenoviruses and poxviruses, are being increasingly tested across various conditions, including SARS‐CoV‐2 and malaria, due to their ability to express multiple genes encoding desired viral antigens through gene recombination. This mechanism allows for the stimulation of both innate and adaptive immunity via cell transduction. The often‐attenuated nature of these viral vectors serves as an immunogen, enhancing host responses and increasing vaccine efficacy by mimicking natural infection.^109^, ^110^ One of the significant advantages of these vaccines is the relative ease of producing and engineering them to carry multiple viral genes, along with the potential for active targeting to restrict the recombinant virus's activity to specific tissues, such as tumors. However, challenges persist, particularly concerning the risk of reversion, where the recombinant vaccine may lose the desired antigens, become more virulent, or induce immunity that limits the virus's ability to infect cells and express pathogen antigens through MHC pathways.^109^, ^110^

An intriguing aspect of vector‐based vaccines is the prime‐boost strategy, where an initial vaccination is followed by boosters using either homologous or heterologous vectors to deliver the same antigen. Homologous boosters can enhance humoral responses, while heterologous vectors can improve Th1 and memory T cell responses, which are particularly beneficial in HIV‐1 treatment.^111^, ^112^ Additionally, combining adenovirus‐vectored vaccines with other vaccine types, such as mRNA or subunit vaccines, has been shown to further enhance humoral responses, as seen in studies involving SARS‐CoV‐2. It is increasingly recognized that the innate immune system's cells, especially macrophages, are trained by the initial vaccination to respond more effectively to subsequent booster doses, leading to improved on‐site and nonspecific immune protection through increased cytokine expression, epigenetic changes, and prolonged activation.^111^, ^112^ This vaccine delivery system holds promise for enhancing vaccine responses in immunocompromised individuals and improving both innate and adaptive immunity against HIV infection, potentially aborting the infection altogether.

An encouraging avenue in the pursuit of an HIV vaccine revolves around exploring bnAbs as potential immune correlates of protection. These specialized antibodies exhibit a distinctive capability to recognize and neutralize a diverse range of HIV variants, primarily owing to their specificity for conserved regions within the virus. Interestingly, however, while efficacious, they are produced in only a minority of HIV‐I‐infected individuals and, when present, are underrepresented.^26^ The prospect of bnAbs offering protection against a spectrum of HIV strains adds significant interest to their investigation as well as evidence that *rhesus macaques* can reduce HIV‐1 DNA levels in gastrointestinal mucosa and lymph nodes and thus the HIV reservoir.^113^ Ongoing research endeavors are dedicated to unraveling the mechanisms governing the development of bnAbs and investigating methods to induce their production through vaccination.^114^, ^115^

Building on the difficulties of eliciting bnAbs against HIV‐1, the second option for potential vaccine targets is bnAbs that prevent virions from entering host cells, thereby preventing HIV integration into the genome. These Env‐specific bnAbs, which are critical for blocking the early stages of infection, undergo extensive somatic mutation within the germinal center. This process may include insertions or deletions in the immunoglobulin heavy and light chains, particularly in the third heavy‐chain complementarity‐determining region (HCDR3) loop, which allows the antibody to effectively combat the protective glycan shield that surrounds the HIV‐1 envelope.^26^, ^116^ It is for this reason that the induction of bnAbs is challenging in vivo.

The breakthrough in bnAb synthesis was made possible by high throughput single‐cell B‐cell receptor amplification assays. Notable antibodies resulting from these efforts, such as 3BNC117 and VRC01, were classified by Scheid as potent antibodies that mimic CD4 binding, effectively acting as a highly active agonist CD4‐binding site. This discovery paved the way for clinical trials to investigate the effect of passive infusions of such bnAbs on people with HIV‐1 infections.^117^, ^118^

In a study, Caskey et al. examined the impact of the antibody 3BNC117, an anti‐CD4 antibody isolated from an elite controller, on the viral load of HIV‐1 infected individuals not receiving ART.^117^ Utilizing a single intravenous dose of between 1 and 30 mg/kg a dose‐effect relationship was noted in viral load with up to a 2.5 log_10_ decline noted (average 1.48 log_10_) and sustained over 28 days. However, a decrease in sensitivity to 3BNC117 (up to fivefold) was observed by day 28 in cultured mononuclear cells.^72^ Similar to this, Lynch et al. carried out a parallel trial involving individuals on and off ART using the VRC01 antibody infusion, which saw modest decreases in viral load (1.1–1.8 log_10_), selection of less sensitive virus, however, instances of primary resistance and reduced benefit on the prescription of a second dose was observed.^118^ This finding was confirmed when in chronically infected and suppressed individuals who had been on ART for 2 years, VRC01 given as two IV doses 40 mg/kg demonstrated no significant reduction in cell‐associated HIV‐I or plasma viremia.^119^ It would seem that these agents may, therefore, have a possible role as an adjuvant therapy helping to control disease flares but cannot in themselves be the primary form of disease management. It may be using intelligent computer‐aided mutational/cell phenotype analysis that design of efficacious bnAb's may be made in the future.

Additional vaccine targets include the V3‐glycan patch and gp41's membrane proximal external region (MPER), which is the target of several bnAbs with liposomal MPER used to produce neutralizing antibodies, albeit with limited success.^120^ Ongoing research focuses on developing immunogens that increase accessibility and elicit antibodies against these regions. Experimental work in *rhesus macaques* has investigated the possibility of inducing HIV‐1‐reactive CD8+ T cells, implying that T cell responses play a role in preventing the formation of a long‐term latent pool of infected CD4+ T cells. This comprehensive approach to vaccine development includes novel platforms such as nucleoside‐modified mRNAs in lipid nanoparticles, emphasizing a determination to overcome obstacles and achieve the goal of inducing protective immune responses against HIV‐1.^26^

A pivotal focus in HIV vaccine development is the viral envelope glycoprotein gp120, which plays a crucial role in the early stages of infection by facilitating viral entry into host cells. Gp120 serves as the primary target for neutralizing antibodies, making it a vital element for vaccine design aimed at eliciting broad neutralizing responses. However, the variability of gp120 poses a substantial challenge, acting as a shield that can impede immune recognition. Navigating this variability is central to vaccine strategies, underscoring the need to effectively stimulate the immune system to produce antibodies capable of neutralizing the diverse spectrum of HIV strains.^121^, ^122^, ^123^

Another tested target for HIV vaccines is the regulatory protein TAT, which is crucial for HIV‐I transcription and replication. TAT is the first protein transcribed from integrated HIV‐1. It interacts with host histone and factor associated acetyltransferases to facilitate viral proviral integration, the phosphorylation of RNA polymerase II and its binding to the viral promoter in the 5' LTR alongside elements of the TATA box while also promoting elongation of HIV transcripts via host positive transcription elongation factor‐B. Without TAT HIV‐1, transcription is 100‐fold reduced, making HIV‐1 infection otherwise unsustainable. Importantly, even in virologically suppressed patients, TAT is detectable within the CSF. Indeed, TAT functions as a messenger molecule when secreted extracellularly where it exhibits immunosuppressive and cytopathic actions of nearby lymphocytes and neuronal cells potentiating onward cell transmission, inflammation and in HIV‐1 patients neurotoxicity and HIV‐1 associated neurocognitive disorders.^124^, ^125^, ^126^

Targeting TAT in vaccine development seeks to disrupt the virus's capacity to replicate and propagate within the host. By stimulating the production of antibodies or cellular immune responses against TAT, researchers aim to disrupt the virus's life cycle, potentially reducing its overall impact on the immune system.^127^, ^128^ Early evidence of this approach shows targeting TAT can improve immune restoration and, when produced in HIV‐I positive patients, is associated with slower disease progression. In those patients in whom TAT‐based vaccination was performed, increased memory CD4 and CD8 cells were observed alongside increased proviral DNA decay in animal models which may abrogate initial infection making this an attractive target for future vaccine design.^128^

Shifting focus from antibody‐centric approaches to cellular immune responses, it becomes essential to consider the role of cytotoxic T lymphocytes (CTLs).^129^ These cells play a critical role in recognizing and eliminating infected cells through MHC I, providing an additional layer of defense in the immune response against HIV. Indeed, HLA‐B*5701 and B*5703 are among the key factors in determining the viral load in infected patients with CTL escape variants seen in chronic infection.^130^ The use of myeloid dendritic cell vaccines to produce highly potent CTLs against an array of HIV‐I epitopes has proven to be a viable approach.^131^ Identifying, therefore, conserved regions of the virus capable of eliciting robust CTL responses is fundamental to developing comprehensive HIV vaccine strategies, ensuring a holistic approach to immune protection.^132^, ^133^ Recently, the use of cytomegalovirus (CMV) as a means to achieve HIV‐I immunity has been proposed given the obvious similarities they share including their ability to produce potent T cell responses and yet persist in the human host indefinitely. Using rhesus CMV vector utilizing SIV antigens both specific and highly active CD4 and CD8 T cell responses presented by MHC‐II, MHC‐E, and MHC‐Ib were produced and were able to functionally cure rhesus monkeys from their SIV in what appears an IL‐5 dependent manner. This is a highly promising finding and has practicalities in CMV seropositive individuals, given that CMV can cause superinfections.^134^

While mRNA vaccines have shown their effectiveness in SARS‐CoV2, it is hoped they could be utilized in HIV. mRNA vaccines have several inherent advantages, including that they are scalable and cheaper than other vaccine types and safer given that they do not risk the development of a “live” virus and, unlike DNA vaccines, cannot alter the host genome. Indeed, the use of mRNA vaccines in SIV has demonstrated profound activity in rhesus monkeys and protection from repeated mucosal challenges.^135^ A key aspect, however, is the need to maintain mRNA stability, which can be achieved by a drug‐delivery platform to ensure their uptake, stability, and immunogenicity. A recent carbon‐nanotube‐based nanoparticle (Nano‐Vac) was utilized to deliver HIV‐I mRNA encoding V1V2 glycoprotein or HIV‐I antigen delivery in a mouse model. While 3‐month stability of mRNA within the Nano‐vac platform was achieved at refrigerator conditions eliminating the need for a cold chain with tolerability demonstrated at high doses (20–30 mg/kg) in rats after 2 weeks, in HIS‐mice there was evidence of durable and strong humoral and cellular immune responses to HIV‐I mRNA with clearance of HIV‐I in some mice.^136^ A recent study evaluated a multiepitope mRNA‐liposomal nanoparticles targeting viral protease cleavage sites, which are conserved between HIV subtypes, has shown great promise by promoting CD8+ T cell activity and memory with minimal CD4+ T cell involvement and inflammation, leading to the possibility of abortion of early infection.^125^ As such, this represents a viable method of managing HIV‐I in the future although the long‐term effects of such nanoparticles are an important consideration given their unique physicochemical properties and the possibility of limited metabolism.

## 7 LIMITATIONS ASSOCIATED WITH CELL AND ANIMAL MODELS

The pursuit of an effective HIV vaccine faces significant impediments, primarily arising from the constraints posed by cell and animal models. These complexities present formidable hurdles in the development of a truly representative and efficacious vaccine. New technologies, such as computational modeling and in silico vaccine design, may assist greatly allowing for rational vaccine design through the identification of putative biomarkers for vaccine development, and allow for estimation of vaccine immunogenicity and pharmacokinetics, potentially saving years in time and considerable money.^137^

A major constraint arises in the application of cell models for HIV research. While traditional cell cultures offer insights into specific aspects of viral replication, they fall short of capturing the intricacies of the human immune system and the dynamic nature of HIV infection between cell types that do not exist within a vacuum. The virus's rapid mutation, coupled with the selective pressures imposed by cell cultures, may not faithfully replicate the in vivo conditions within a human host. Consequently, findings from cell models often struggle to translate effectively to human responses, introducing complications in predicting vaccine efficacy.^138^, ^139^

In the domain of preclinical studies, animal models are indispensable, allowing for HIV‐1 infection to be studied at all stages and bodily compartments, e.g., blood, CSF, and bone marrow, in an ethically correct way. Of animal models, SIV infection in nonhuman primates, particularly *rhesus macaques*, being a common choice.^140^ However, SIV does not perfectly mimic HIV in humans, posing challenges in the translatability of results. Notably, African SIV exhibits disparities in disease progression compared to HIV in humans, including a prolonged latency period in SIV‐infected *rhesus macaques* as opposed to the comparatively shorter timeframe observed in HIV‐infected humans. This prolonged latency complicates the evaluation of vaccine efficacy and the projection of long‐term protective immunity.^141^ Moreover, underlying genetic and physiological differences between nonhuman primates and humans makes comparison even harder. Although chimpanzees represent the closest genetic model and may be naturally HIV‐1 infected, they rarely develop AIDS with little CD4 decline and innately less impactful CD8 responses versus humans, meaning they are seldom used. In contrast, *rhesus macaques* and SIV utilize different chemokine receptors but may faithfully infect resting and activated CD4 T cells, albeit with differences in pathogenicity between SIV strains.^142^ Feline immunodeficiency virus, while common in domestic cats like HIV, results in progressive CD4 decline, although demonstrates a lower evolution rate and can take a decade to reach the chronic phase limiting the utility of this model.^139^, ^143^

The genetic divergence between SIV and HIV presents further complications in extrapolating findings from SIV studies to humans. Despite shared similarities, differences in viral tropism, disease progression, and immune responses necessitate caution when interpreting results from SIV studies. The intricate interplay between HIV and the human immune system, encompassing aspects such as the establishment of latent viral reservoirs and the virus's ability to evade immune detection, may not be accurately replicated in SIV‐infected macaques.^144^, ^145^

Indeed, this is a key issue with animal models, which must have the necessary cellular infrastructure to support HIV replication, e.g., CD4 and CCR5. For instance, while for example domestic rabbits procreate quickly and cost little to maintain, they cannot sustain HIV‐1 infection necessitating transgenic models to be made.^114^ While murine models are used and may be infected with HIV‐1 and share similar infection characteristics to be faithful, they require to be humanized. To do this, there must be strain selection of mice with chemoirradiation to ablate their immune system to facilitate the subsequent transplantation of human fetal/haemopoietic stem cells or tissue (thymus/liver) into the mice and their sequential engraftment and immune reconstitution over several months.^146^ This process, while useful, can lead to only limited immune system development depending on the method employed (e.g., SCID‐hu mice and no primary immune response), while others generate a naïve immune system that does not clearly depict the interactions between HIV‐1 and the human host. This process leads to restricted viraemia and colonization and will lead to eventual graft‐versus host disease, which limits the duration of observation.^146^ While limitations exist, murine models, in particular the Hu‐mouse model, have proven useful in examining mucosal transmission of HIV‐1 and the effect the microbiome has on this risk, as well as tuberculosis‐HIV infection where immune reconstitution may occur.^147^ Moreover, such humanized models allow for the proper evaluation of antiviral drugs and new insights into the effects of HIV‐1 mediated chronic immune activation. To this end, humanized rat models may be the preferred animal model going forward given their longer lifespans, size, and cognitive/physiological likeness to humans versus mouse models from which HIV‐specific organ disease could be studied and vaccine targets investigated.^148^

A pivotal challenge emerges in the impossibility of conducting vaccine trials with HIV, primarily due to ethical and safety concerns.^149^, ^150^ Unlike diseases where live attenuated or inactivated vaccines are standard, devising a safe and effective HIV vaccine has proven exceptionally challenging. The associated risks of using live attenuated HIV, even in a weakened state, are considerable, considering the potential for reversion to a more virulent form. Conversely, inactivated vaccines may fall short of inducing the robust and enduring immune responses required for effective HIV prevention.^151^, ^152^

## 8 CONCLUSION

Despite decades of research since the isolation of HIV‐1 and the development of effective treatments, the virus remains a formidable force, causing substantial mortality and morbidity worldwide. Studies over the years have significantly advanced our understanding of HIV‐1's viral kinetics, host susceptibility, and antiviral immunity, leading to broader advancements in infectious disease diagnosis and management. However, HIV‐1's persistence can be attributed to its remarkable ability to infiltrate various bodily compartments, establish viral reservoirs, manipulate and suppress immune responses, and integrate its genetic material into the host genome. While the exact factors conferring immune protection against HIV‐1 are not fully understood, it is evident that robust CTL responses and bnAbs provide substantial advantages, albeit with challenges in their production and control. Innovative approaches, such as leveraging computational learning to comprehend mutation pressures and employing advanced drug delivery systems like viral vectors and nanoparticles, offer promising avenues. These strategies aim to enhance the activation of the immune system, protecting the development of viral reservoirs and possibly preventing infection. While substantial progress has been achieved in understanding the pathophysiology of HIV‐1, identifying correlates of protection, and designing effective vaccines, it is important to maintain ongoing investment in research. This includes addressing the discrepancies between animal and human models and exploring combination approaches that, alongside improved access to ART, offer the potential to ultimately end the HIV‐1 pandemic. Continued efforts in these areas are essential for developing comprehensive strategies to combat the virus effectively.

## AUTHOR CONTRIBUTIONS

**Godfred Yawson Scott**: Conceptualization; writing—original draft; writing—review and editing; visualization; validation; methodology; software; formal analysis. **Dominic Worku**: Conceptualization; investigation; visualization; validation; methodology; writing—review and editing; writing—original draft; project administration; formal analysis; supervision.

## CONFLICTS OF INTEREST STATEMENT

The authors declare no conflict of interest.

## TRANSPARENCY STATEMENT

The lead author Godfred Yawson Scott affirms that this manuscript is an honest, accurate, and transparent account of the study being reported; that no important aspects of the study have been omitted; and that any discrepancies from the study as planned (and, if relevant, registered) have been explained.

## Data Availability

Data sharing is not applicable to this article as no datasets were generated or analyzed during the current study.

## References

[1] j0294‐who‐hiv‐epi‐factsheet‐v7.pdf. 2023. Accessed February 13, 2024. https://cdn.who.int/media/docs/default-source/hq-hiv-hepatitis-and-stis-library/j0294-who-hiv-epi-factsheet-v7.pdf

[2] Global HIV Programme. 2024. Accessed February 13, 2024. 2024. Accessed February 13, https://www.who.int/teams/global-hiv-hepatitis-and-stis-programmes/hiv/strategic-information/hiv-data-and-statistics

[3] Local Burden of Disease HIV Collaborators . Subnational mapping of HIV incidence and mortality among individuals aged 15–49 years in sub‐Saharan Africa, 2000–18: a modelling study. Lancet HIV. 2021;8(6):e363‐e375. 34087097 10.1016/S2352-3018(21)00051-5 PMC8187986

[4] Mahy MI , Sabin KM , Feizzadeh A , Wanyeki I . Progress towards 2020 global HIV impact and treatment targets. J Int AIDS Soc. 2021;24(suppl 5):e25779. 34546655 10.1002/jia2.25779 PMC8454678

[5] Frescura L , Godfrey‐Faussett P , Feizzadeh A. A , et al. Achieving the 95 95 95 targets for all: a pathway to ending AIDS. PLoS One. 2022;17(8):e0272405. 35925943 10.1371/journal.pone.0272405 PMC9352102

[6] Marsh K , Eaton JW , Mahy M , et al. Global, regional and country‐level 90‐90‐90 estimates for 2018: assessing progress towards the 2020 target. AIDS. 2019;33:S213‐S226. 31490781 10.1097/QAD.0000000000002355 PMC6919229

[7] Heath K , Levi J , Hill A . The Joint United Nations Programme on HIV/AIDS 95‐95‐95 targets: worldwide clinical and cost benefits of generic manufacture. AIDS. 2021;35(suppl 2):S197‐S203. 34115649 10.1097/QAD.0000000000002983

[8] Payne D , Wadonda‐Kabondo N , Wang A , et al. Trends in HIV prevalence, incidence, and progress towards the UNAIDS 95‐95‐95 targets in Malawi among individuals aged 15–64 years: population‐based HIV impact assessments, 2015−16 and 2020−21. Lancet HIV. 2023;10(9):e597‐e605. 37586390 10.1016/S2352-3018(23)00144-3 PMC10542580

[9] HIV prevention: from crisis to opportunity—Key findings from the 2023 Global HIV Prevention Coalition scorecards. 2024. Accessed Aug 1, 2024. 2024. Accessed Aug 1, https://www.unaids.org/en/resources/documents/2024/2023-global-hiv-prevention-coalition-scorecards-key-findings

[10] Maheu‐Giroux M , Mishra S . Evidence with 95‐95‐95 that ambitious is feasible. Lancet HIV. 2024;11(4):e203‐e204. 38467134 10.1016/S2352-3018(24)00028-6

[11] Marcus JL , Leyden WA , Alexeeff SE , et al. Comparison of overall and comorbidity‐free life expectancy between insured adults with and without HIV infection, 2000–2016. JAMA Network Open. 2020;3(6):e207954. 32539152 10.1001/jamanetworkopen.2020.7954 PMC7296391

[12] Moreno S , Perno C , Mallon P , et al. Two‐drug vs. three‐drug combinations for HIV‐1: do we have enough data to make the switch? HIV Med. 2019;20(suppl 4):2‐12. 10.1111/hiv.12716 30821898

[13] Angus B , Gary B , Funmi A . BHIVA guidelines for the routine investigation and monitoring of adult HIV‐1‐positive individuals (2019 interim update). https://www.bhiva.org/monitoring-guidelines

[14] Freiberg MS , So‐Armah K . HIV and cardiovascular disease: we need a mechanism, and we need a plan. J Am Heart Assoc. 2016;5(3):e003411. 10.1161/JAHA.116.003411 PMC4943288 27013540

[15] Ergin HE , Inga EE , Maung TZ , Javed M , Khan S . HIV, antiretroviral therapy and metabolic alterations: a review. Cureus. 2020;12(5):e8059. 32537277 10.7759/cureus.8059 PMC7286589

[16] Edwards GG , Miyashita‐Ochoa A , Castillo EG , et al. Long‐acting injectable therapy for people with HIV: looking ahead with lessons from psychiatry and addiction medicine. AIDS Behav. 2023;27(1):10‐24. 36063243 10.1007/s10461-022-03817-z PMC9443641

[17] Whiteley LB , Olsen EM , Haubrick KK , Odoom E , Tarantino N , Brown LK . A review of interventions to enhance HIV medication adherence. Curr HIV/AIDS Rep. 2021;18(5):443‐457. 34152554 10.1007/s11904-021-00568-9 PMC12166660

[18] Kim J , Lee E , Park BJ , Bang JH , Lee JY . Adherence to antiretroviral therapy and factors affecting low medication adherence among incident HIV‐infected individuals during 2009–2016: a nationwide study. Sci Rep. 2018;8(1):3133. 29453393 10.1038/s41598-018-21081-x PMC5816616

[19] Kim YS . Long‐acting injectable antiretroviral agents for HIV treatment and prevention. Infect Chemother. 2021;53(4):686‐695. 34979604 10.3947/ic.2021.0136 PMC8731252

[20] Wang W , Zhao S , Wu Y , et al. Safety and efficacy of long‐acting injectable agents for HIV‐1: systematic review and meta‐analysis. JMIR Public Health Surveill. 2023;9:e46767. 37498645 10.2196/46767 PMC10415942

[21] Fonner VA , Ridgeway K , van der Straten A , et al. Safety and efficacy of long‐acting injectable cabotegravir as preexposure prophylaxis to prevent HIV acquisition. AIDS. 2023;37(6):957‐966. 36723489 10.1097/QAD.0000000000003494 PMC10090368

[22] Jolayemi O , Bogart LM , Storholm ED , et al. Perspectives on preparing for long‐acting injectable treatment for HIV among consumer, clinical and nonclinical stakeholders: a qualitative study exploring the anticipated challenges and opportunities for implementation in Los Angeles County. PLoS One. 2022;17(2):e0262926. 35113892 10.1371/journal.pone.0262926 PMC8812879

[23] Greenwood B . The contribution of vaccination to global health: past, present and future. Philos Trans R Soc, B. 2014;369(1645):20130433. 10.1098/rstb.2013.0433 PMC4024226 24821919

[24] Barouch DH . Challenges in the development of an HIV‐1 vaccine. Nature. 2008;455(7213):613‐619. 18833271 10.1038/nature07352 PMC2572109

[25] Trougakos IP , Terpos E , Alexopoulos H , et al. Adverse effects of COVID‐19 mRNA vaccines: the spike hypothesis. Trends Mol Med. 2022;28(7):542‐554. 35537987 10.1016/j.molmed.2022.04.007 PMC9021367

[26] Haynes BF , Wiehe K , Borrow P , et al. Strategies for HIV‐1 vaccines that induce broadly neutralizing antibodies. Nat Rev Immunol. 2023;23(3):142‐158. 35962033 10.1038/s41577-022-00753-w PMC9372928

[27] de Montigny S , Adamson BJS , Mâsse BR , et al. Projected effectiveness and added value of HIV vaccination campaigns in South Africa: a modeling study. Sci Rep. 2018;8(1):6066. 29666455 10.1038/s41598-018-24268-4 PMC5904131

[28] Arhel N . Revisiting HIV‐1 uncoating. Retrovirology. 2010;7:96. https://pubmed.ncbi.nlm.nih.gov/21083892/ 21083892 10.1186/1742-4690-7-96 PMC2998454

[29] Campbell EM , Hope TJ . HIV‐1 capsid: the multifaceted key player in HIV‐1 infection. Nat Rev Microbiol. 2015;13(8):471‐483. 26179359 10.1038/nrmicro3503 PMC4876022

[30] Deeks SG , Overbaugh J , Phillips A , Buchbinder S . HIV infection. Nat Rev Dis Primer. 2015;1:15035. https://pubmed.ncbi.nlm.nih.gov/27188527/ 10.1038/nrdp.2015.35 27188527

[31] Mailler M , Bernacchi S , Marquet R , Paillart J‐C , Vivet‐Boudou V , Smyth RP . The life‐cycle of the HIV‐1 Gag‐RNA complex. Viruses. 2016;8(9):248. https://pubmed.ncbi.nlm.nih.gov/27626439/ 27626439 10.3390/v8090248 PMC5035962

[32] Freed EO . HIV‐1 assembly, release and maturation. Nat Rev Microbiol. 2015;13(8):484‐496. https://pubmed.ncbi.nlm.nih.gov/26119571/ 26119571 10.1038/nrmicro3490 PMC6936268

[33] Goodsell DS . Illustrations of the HIV life cycle. Curr Top Microbiol Immunol. 2015;389:243‐252. https://pubmed.ncbi.nlm.nih.gov/25716304/ 25716304 10.1007/82_2015_437

[34] Jasinska AJ , Pandrea I , Apetrei C . CCR5 as a coreceptor for human immunodeficiency virus and simian immunodeficiency viruses: a prototypic love‐hate affair. Front Immunol. 2022;13:835994. 35154162 10.3389/fimmu.2022.835994 PMC8829453

[35] Alkhatib G . The biology of CCR5 and CXCR4. Current Opinion in HIV and AIDS. 2009;4(2):96‐103. 19339947 10.1097/COH.0b013e328324bbec PMC2718543

[36] HIV Replication Cycle | NIH: National Institute of Allergy and Infectious Diseases. 2018. Accessed November 21, 2023. https://www.niaid.nih.gov/diseases-conditions/hiv-replication-cycle

[37] Negi G , Sharma A , Dey M , Dhanawat G , Parveen N . Membrane attachment and fusion of HIV‐1, influenza A, and SARS‐CoV‐2: resolving the mechanisms with biophysical methods. Biophys Rev. 2022;14(5):1109‐1140. https://pubmed.ncbi.nlm.nih.gov/36249860/ 36249860 10.1007/s12551-022-00999-7 PMC9552142

[38] Xiao T , Cai Y , Chen B . HIV‐1 entry and membrane fusion inhibitors. Viruses. 2021;13(5):735. https://pubmed.ncbi.nlm.nih.gov/33922579/ 33922579 10.3390/v13050735 PMC8146413

[39] Sapp N , Burge N , Cox K , et al. HIV‐1 preintegration complex preferentially integrates the viral DNA into nucleosomes containing trimethylated histone 3‐lysine 36 modification and flanking linker DNA. J Virol. 2022;96(18):e0101122. 36094316 10.1128/jvi.01011-22 PMC9517705

[40] Rozina A , Anisenko A , Kikhai T , Silkina M , Gottikh M . Complex relationships between HIV‐1 integrase and its cellular partners. Int J Mol Sci. 2022;23(20):12341. 36293197 10.3390/ijms232012341 PMC9603942

[41] Nielsen M , Pedersen F , Kjems J . Molecular strategies to inhibit HIV‐1 replication. Retrovirology. 2005;2(1):10. 15715913 10.1186/1742-4690-2-10 PMC553987

[42] Ebina H , Misawa N , Kanemura Y , Koyanagi Y . Harnessing the CRISPR/Cas9 system to disrupt latent HIV‐1 provirus. Sci Rep. 2013;3(1):2510. 23974631 10.1038/srep02510 PMC3752613

[43] Zhang X , Ma X , Jing S , Zhang H , Zhang Y . Non‐coding RNAs and retroviruses. Retrovirology. 2018;15(1):20. 29426337 10.1186/s12977-018-0403-8 PMC5807749

[44] The HIV Life Cycle | NIH. 2021. Accessed November 23, 2023. 2021. Accessed November 23, 2023. https://hivinfo.nih.gov/understanding-hiv/fact-sheets/hiv-life-cycle

[45] Shcherbatova O , Grebennikov D , Sazonov I , Meyerhans A , Bocharov G . Modeling of the HIV‐1 life cycle in productively infected cells to predict novel therapeutic targets. Pathogens. 2020;9(4):255. 32244421 10.3390/pathogens9040255 PMC7238236

[46] Sundquist WI , Krausslich HG . HIV‐1 assembly, budding, and maturation. Cold Spring Harbor Perspect Med. 2012;2(7):a006924. 10.1101/cshperspect.a006924 PMC3385941 22762019

[47] Klingler J , Anton H , Réal E , et al. How HIV‐1 gag manipulates its host cell proteins: a focus on interactors of the nucleocapsid domain. Viruses. 2020;12(8):888. 32823718 10.3390/v12080888 PMC7471995

[48] Burniston MT , Cimarelli A , Colgan J , Curtis SP , Luban J . Human immunodeficiency virus type 1 gag polyprotein multimerization requires the nucleocapsid domain and RNA and is promoted by the capsid‐dimer interface and the basic region of matrix protein. J Virol. 1999;73(10):8527‐8540. 10482606 10.1128/jvi.73.10.8527-8540.1999 PMC112873

[49] Hendricks CM , Cordeiro T , Gomes AP , Stevenson M . The interplay of HIV‐1 and macrophages in viral persistence. Front Microbiol. 2021;12:646447. 33897659 10.3389/fmicb.2021.646447 PMC8058371

[50] Delannoy A , Poirier M , Bell B . Cat and mouse: HIV transcription in latency, immune evasion and cure/remission strategies. Viruses. 2019;11(3):269. 30889861 10.3390/v11030269 PMC6466452

[51] Lu L , Yu F , Du L , Xu W , Jiang S . Tactics used by HIV‐1 to evade host innate, adaptive, and intrinsic immunities. Chin Med J. 2013;126(12):2374‐2379. 23786957

[52] Balasubramaniam M , Pandhare J , Dash C . Immune control of HIV. J Life Sci. 2019;1(1):4‐37. PMC6714987 31468033

[53] Vidya Vijayan KK , Karthigeyan KP , Tripathi SP , Hanna LE . Pathophysiology of CD4+ T‐cell depletion in HIV‐1 and HIV‐2 infections. Front Immunol. 2017;8:580. 28588579 10.3389/fimmu.2017.00580 PMC5440548

[54] Abu‐Raya B , Kollmann TR , Marchant A , MacGillivray DM . The immune system of HIV‐exposed uninfected infants. Front Immunol. 2016;7:383. https://www.frontiersin.org/articles/10.3389/fimmu.2016.00383 27733852 10.3389/fimmu.2016.00383 PMC5039172

[55] Casper C , Crane H , Menon M , Money D . HIV/AIDS comorbidities: impact on Cancer, noncommunicable diseases, and reproductive health. In: Holmes KK , Bertozzi S , Bloom BR , Jha P , eds. Major Infectious Diseases. 3rd ed. The International Bank for Reconstruction and Development/The World Bank; 2017. 30212097

[56] Boasso A , Shearer GM , Chougnet C . Immune dysregulation in human immunodeficiency virus infection: know it, fix it, prevent it? J Intern Med. 2009;265(1):78‐96. 19093962 10.1111/j.1365-2796.2008.02043.x PMC2903738

[57] Okoye AA , Picker LJ . CD4(+) T‐cell depletion in HIV infection: mechanisms of immunological failure. Immunol Rev. 2013;254(1):54‐64. 23772614 10.1111/imr.12066 PMC3729334

[58] Février M , Dorgham K , Rebollo A . CD4+ T cell depletion in human immunodeficiency virus (HIV) infection: role of apoptosis. Viruses. 2011;3(5):586‐612. 21994747 10.3390/v3050586 PMC3185763

[59] Mawle AC , Mcdougal JS . Immunology of HIV Infection. In: Schochetman G , George JR , eds. AIDS Testing. Springer New York; 1994:32‐51.

[60] Borrell M , Fernández I , Etcheverrry F , et al. High rates of long‐term progression in HIV‐1‐positive elite controllers. J Int AIDS Soc. 2021;24(2):e25675. 33619912 10.1002/jia2.25675 PMC7900439

[61] Saag M , Deeks SG . How do HIV elite controllers do what they do? Clin Infect Dis. 2010;51(2):239‐241. 20550453 10.1086/653678

[62] Gebara NY , El Kamari V , Rizk N . HIV‐1 elite controllers: an immunovirological review and clinical perspectives. J Virus Erad. 2019;5(3):163‐166. 31700663 10.1016/S2055-6640(20)30046-7 PMC6816117

[63] Goulder PJR , Watkins DI . Impact of MHC class I diversity on immune control of immunodeficiency virus replication. Nat Rev Immunol. 2008;8(8):619‐630. 18617886 10.1038/nri2357 PMC2963026

[64] Hewson T , Lone N , Moore M , Howie S . Interactions of HIV‐1 with antigen‐presenting cells. Immunol Cell Biol. 1999;77(4):289‐303. 10457195 10.1046/j.1440-1711.1999.00833.x

[65] Fanales‐Belasio E , Raimondo M , Suligoi B , Buttò S . HIV virology and pathogenetic mechanisms of infection: a brief overview. Annali dell'Istituto superiore di sanita. 2010;46:5‐14. 10.4415/ANN_10_01_02 20348614

[66] Koyanagi N , Kawaguchi Y . Evasion of the cell‐mediated immune response by alphaherpesviruses. Viruses. 2020;12(12):1354. 33256093 10.3390/v12121354 PMC7761393

[67] Stumptner‐Cuvelette P , Morchoisne S , Dugast M , et al. HIV‐1 Nef impairs MHC class II antigen presentation and surface expression. Proc Natl Acad Sci. 2001;98(21):12144‐12149. 11593029 10.1073/pnas.221256498 PMC59782

[68] Staudt RP , Alvarado JJ , Emert‐Sedlak LA , et al. Structure, function, and inhibitor targeting of HIV‐1 Nef‐effector kinase complexes. J Biol Chem. 2020;295(44):15158‐15171. 32862141 10.1074/jbc.REV120.012317 PMC7606690

[69] Wonderlich ER , Leonard JA , Collins KL . HIV immune evasion disruption of antigen presentation by the HIV Nef protein. Adv Virus Res. 2011;80:103‐127. 21762823 10.1016/B978-0-12-385987-7.00005-1 PMC3782996

[70] Roeth JF , Collins KL . Human immunodeficiency virus type 1 Nef: adapting to intracellular trafficking pathways. Microbiol Mol Biol Rev. 2006;70(2):548‐563. 16760313 10.1128/MMBR.00042-05 PMC1489538

[71] Pereira EA , daSilva LLP . HIV‐1 nef: taking control of protein trafficking. Traffic. 2016;17(9):976‐996. 27161574 10.1111/tra.12412

[72] Buffalo CZ , Iwamoto Y , Hurley JH , Ren X . How HIV nef proteins hijack membrane traffic to promote infection. J Virol. 2019;93(24):e01322‐19. 31578291 10.1128/JVI.01322-19 PMC6880166

[73] Blagoveshchenskaya AD , Thomas L , Feliciangeli SF , Hung CH , Thomas G . HIV‐1 Nef downregulates MHC‐I by a PACS‐1‐ and PI3K‐regulated ARF6 endocytic pathway. Cell. 2002;111(6):853‐866. 12526811 10.1016/s0092-8674(02)01162-5

[74] Dubé M , Bego MG , Paquay C , Cohen ÉA . Modulation of HIV‐1‐host interaction: role of the Vpu accessory protein. Retrovirology. 2010;7:114. 21176220 10.1186/1742-4690-7-114 PMC3022690

[75] Khan N , Geiger JD . Role of viral protein U (Vpu) in HIV‐1 infection and pathogenesis. Viruses. 2021;13(8):1466. 34452331 10.3390/v13081466 PMC8402909

[76] Mitchell RS , Katsura C , Skasko MA , et al. Vpu antagonizes BST‐2‐mediated restriction of HIV‐1 release via β‐TrCP and endo‐lysosomal trafficking. PLoS Pathog. 2009;5(5):e1000450. 19478868 10.1371/journal.ppat.1000450 PMC2679223

[77] Le Tortorec A , Willey S , Neil SJD . Antiviral inhibition of enveloped virus release by tetherin/BST‐2: action and counteraction. Viruses. 2011;3(5):520‐540. 21994744 10.3390/v3050520 PMC3185764

[78] Tetherin antagonism by Vpu protects HIV‐infected cells from antibody‐dependent cell‐mediated cytotoxicity ‐ PubMed. 2014. Accessed November 24, 2023. https://pubmed.ncbi.nlm.nih.gov/24733916/ 10.1073/pnas.1321507111 PMC4035966 24733916

[79] Lata S , Mishra R , Banerjea AC . Proteasomal degradation machinery: favorite target of HIV‐1 proteins. Front Microbiol. 2018;9:2738. 30524389 10.3389/fmicb.2018.02738 PMC6262318

[80] Rojas VK , Park IW . Role of the ubiquitin proteasome system (UPS) in the HIV‐1 life cycle. Int J Mol Sci. 2019;20(12):2984. 31248071 10.3390/ijms20122984 PMC6628307

[81] Guha D , Ayyavoo V . Innate immune evasion strategies by human immunodeficiency virus type 1. ISRN AIDS. 2013;2013:1‐10. 10.1155/2013/954806 PMC3767209 24052891

[82] Carrington M , Alter G . Innate immune control of HIV. Cold Spring Harbor Perspect Med. 2012;2(7):a007070. 10.1101/cshperspect.a007070 PMC3385945 22762020

[83] Scholz EMB , Kashuba ADM . The lymph node reservoir: physiology, HIV infection, and antiretroviral therapy. Clin Pharm Ther. 2021;109(4):918‐927. 10.1002/cpt.2186 PMC8005487 33529355

[84] Blankson JN , Persaud D , Siliciano RF . The challenge of viral reservoirs in HIV‐1 infection. Annu Rev Med. 2002;53(1):557‐593. 11818490 10.1146/annurev.med.53.082901.104024

[85] Zhang X , Chen J . HIV reservoir: how to measure it? Curr HIV/AIDS Rep. 2023;20(2):29‐41. 37004676 10.1007/s11904-023-00653-1

[86] Sun W , Gao C , Hartana CA , et al. Phenotypic signatures of immune selection in HIV‐1 reservoir cells. Nature. 2023;614(7947):309‐317. 36599977 10.1038/s41586-022-05538-8 PMC9908552

[87] Chun TW , Stuyver L , Mizell SB , et al. Presence of an inducible HIV‐1 latent reservoir during highly active antiretroviral therapy. Proc Natl Acad Sci. 1997;94(24):13193‐13197. 9371822 10.1073/pnas.94.24.13193 PMC24285

[88] Finzi D , Hermankova M , Pierson T , et al. Identification of a reservoir for HIV‐1 in patients on highly active antiretroviral therapy. Science. 1997;278(5341):1295‐1300. 9360927 10.1126/science.278.5341.1295

[89] Wong JK , Hezareh M , Günthard HF , et al. Recovery of replication‐competent HIV despite prolonged suppression of plasma viremia. Science. 1997;278(5341):1291‐1295. 9360926 10.1126/science.278.5341.1291

[90] Siliciano JD , Kajdas J , Finzi D , et al. Long‐term follow‐up studies confirm the stability of the latent reservoir for HIV‐1 in resting CD4+ T cells. Nat Med. 2003;9(6):727‐728. 12754504 10.1038/nm880

[91] Davey RT , Bhat N , Yoder C , et al. HIV‐1 and T cell dynamics after interruption of highly active antiretroviral therapy (HAART) in patients with a history of sustained viral suppression. Proc Natl Acad Sci. 1999;96(26):15109‐15114. 10611346 10.1073/pnas.96.26.15109 PMC24781

[92] Chomont N , El‐Far M , Ancuta P , et al. HIV reservoir size and persistence are driven by T cell survival and homeostatic proliferation. Nat Med. 2009;15(8):893‐900. 19543283 10.1038/nm.1972 PMC2859814

[93] Chun TW . HIV‐infected individuals receiving effective antiviral therapy for extended periods of time continually replenish their viral reservoir. J Clin Invest. 2005;115(11):3250‐3255. 16276421 10.1172/JCI26197 PMC1265878

[94] Fletcher CV , Staskus K , Wietgrefe SW , et al. Persistent HIV‐1 replication is associated with lower antiretroviral drug concentrations in lymphatic tissues. Proc Natl Acad Sci. 2014;111(6):2307‐2312. 24469825 10.1073/pnas.1318249111 PMC3926074

[95] Cory TJ , Schacker TW , Stevenson M , Fletcher CV . Overcoming pharmacologic sanctuaries. Curr Opin HIV AIDS. 2013;8(3):190‐195. 23454865 10.1097/COH.0b013e32835fc68a PMC3677586

[96] Kimata JT , Rice AP , Wang J . Challenges and strategies for the eradication of the HIV reservoir. Curr Opin Immunol. 2016;42:65‐70. 27288651 10.1016/j.coi.2016.05.015 PMC5086301

[97] Besson GJ , Lalama CM , Bosch RJ , et al. HIV‐1 DNA decay dynamics in blood during more than a decade of suppressive antiretroviral therapy. Clin Infect Dis. 2014;59(9):1312‐1321. 25073894 10.1093/cid/ciu585 PMC4200019

[98] Williams A , Menon S , Crowe M , et al. Geographic and population distributions of human immunodeficiency virus (HIV)‐1 and HIV‐2 circulating subtypes: a systematic literature review and meta‐analysis (2010–2021). J Infect Dis. 2023;228(11):1583‐1591. 37592824 10.1093/infdis/jiad327 PMC10681860

[99] Bbosa N , Kaleebu P , Ssemwanga D . HIV subtype diversity worldwide. Curr Opin HIV AIDS. 2019;14(3):153‐160. 30882484 10.1097/COH.0000000000000534

[100] Santoro MM , Perno CF . HIV‐1 genetic variability and clinical implications. ISRN Microbiol. 2013;2013:481314. 23844315 10.1155/2013/481314 PMC3703378

[101] Götte M , Li X , Wainberg MA . HIV‐1 reverse transcription: a brief overview focused on structure–function relationships among molecules involved in initiation of the reaction. Arch Biochem Biophys. 1999;365(2):199‐210. 10328813 10.1006/abbi.1999.1209

[102] Wei X , Ghosh SK , Taylor ME , et al. Viral dynamics in human immunodeficiency virus type 1 infection. Nature. 1995;373(6510):117‐122. 7529365 10.1038/373117a0

[103] Domingo E , García‐Crespo C , Lobo‐Vega R , Perales C . Mutation rates, mutation frequencies, and proofreading‐repair activities in RNA virus genetics. Viruses. 2021;13(9):1882. 34578463 10.3390/v13091882 PMC8473064

[104] Prado JG , Parkin NT , Clotet B , Ruiz L , Martinez‐Picado J . HIV type 1 fitness evolution in antiretroviral‐experienced patients with sustained CD4+ T cell counts but persistent virologic failure. Clin Infect Dis. 2005;41(5):729‐737. 16080097 10.1086/432619

[105] HIV‐1 Transmission, Replication Fitness and Disease Progression ‐ PMC. 2008. Accessed Aug 1, 2024. 2008. Accessed Aug 1, 2024. https://www.ncbi.nlm.nih.gov/pmc/articles/PMC2846839/ PMC2846839 20354593

[106] Moutouh L , Corbeil J , Richman DD . Recombination leads to the rapid emergence of HIV‐1 dually resistant mutants under selective drug pressure. Proc Natl Acad Sci. 1996;93(12):6106‐6111. 8650227 10.1073/pnas.93.12.6106 PMC39197

[107] Chomont N . Silence, escape and survival drive the persistence of HIV. Nature. 2023;614(7947):236‐237. 36599993 10.1038/d41586-022-04492-9

[108] Naveed M , Ali U , Karobari MI , et al. A vaccine construction against COVID‐19‐associated mucormycosis contrived with immunoinformatics‐based scavenging of potential mucoralean epitopes. Vaccines. 2022;10(5):664. 35632420 10.3390/vaccines10050664 PMC9147184

[109] Wang S , Liang B , Wang W , et al. Viral vectored vaccines: design, development, preventive and therapeutic applications in human diseases. Signal Transduct Target Ther. 2023;8(1):149. 37029123 10.1038/s41392-023-01408-5 PMC10081433

[110] Travieso T , Li J , Mahesh S , Mello JDFRE , Blasi M . The use of viral vectors in vaccine development. NPJ Vaccines. 2022;7(1):1‐10. 35787629 10.1038/s41541-022-00503-y PMC9253346

[111] Palgen JL , Feraoun Y , Dzangué‐Tchoupou G , et al. Optimize prime/boost vaccine strategies: trained immunity as a new player in the game. Front Immunol. 2021;12:612747. 33763063 10.3389/fimmu.2021.612747 PMC7982481

[112] He Q , Mao Q , An C , et al. Heterologous prime‐boost: breaking the protective immune response bottleneck of COVID‐19 vaccine candidates. Emerg Microbes Infect. 2021;10(1):629‐637. 33691606 10.1080/22221751.2021.1902245 PMC8009122

[113] Barouch DH , Whitney JB , Moldt B , et al. Therapeutic efficacy of potent neutralizing HIV‐1‐specific monoclonal antibodies in SHIV‐infected rhesus monkeys. Nature. 2013;503(7475):224‐228. 24172905 10.1038/nature12744 PMC4017780

[114] Kumar R , Qureshi H , Deshpande S , Bhattacharya J . Broadly neutralizing antibodies in HIV‐1 treatment and prevention. Thera Adv Vacc Immunother. 2018;6(4):61‐68. 10.1177/2515135518800689 PMC6187420 30345419

[115] Stephenson KE , Wagh K , Korber B , Barouch DH . Vaccines and broadly neutralizing antibodies for HIV‐1 prevention. Annu Rev Immunol. 2020;38:673‐703. 32340576 10.1146/annurev-immunol-080219-023629 PMC7375352

[116] Hargrave A , Mustafa AS , Hanif A , Tunio JH , Hanif SNM . Current status of HIV‐1 vaccines. Vaccines. 2021;9(9):1026. 34579263 10.3390/vaccines9091026 PMC8471857

[117] Caskey M , Klein F , Lorenzi JCC , et al. Viraemia suppressed in HIV‐1‐infected humans by broadly neutralizing antibody 3BNC117. Nature. 2015;522(7557):487‐491. 25855300 10.1038/nature14411 PMC4890714

[118] Lynch RM , Boritz E , Coates EE , et al. Virologic effects of broadly neutralizing antibody VRC01 administration during chronic HIV‐1 infection. Sci Transl Med. 2015;7(319):319ra206. 10.1126/scitranslmed.aad5752 PMC12366723 26702094

[119] Riddler SA , Zheng L , Durand CM , et al. Randomized clinical trial to assess the impact of the broadly neutralizing HIV‐1 monoclonal antibody VRC01 on HIV‐1 persistence in individuals on effective ART. Open Forum Infect Dis. 2018;5(10):ofy242. 30364428 10.1093/ofid/ofy242 PMC6195652

[120] Shao S , Huang WC , Lin C , et al. An engineered biomimetic MPER peptide vaccine induces weakly HIV neutralizing antibodies in mice. Ann Biomed Eng. 2020;48(7):1991‐2001. 31832930 10.1007/s10439-019-02398-8 PMC7289672

[121] Duarte J . HIV vaccines: gp120 and beyond. Nat Res. 2018. https://www.nature.com/articles/d42859-018-00010-y

[122] Tong T , D'Addabbo A , Xu J , et al. Impact of stabilizing mutations on the antigenic profile and glycosylation of membrane‐expressed HIV‐1 envelope glycoprotein. PLoS Pathog. 2023;19(8):e1011452. 37549185 10.1371/journal.ppat.1011452 PMC10434953

[123] Rao M , Peachman K , Kim J , et al. HIV‐1 variable loop 2 and its importance in HIV‐1 infection and vaccine development. Curr HIV Res. 2013;11(5):427‐438. 24191938 10.2174/1570162x113116660064 PMC4086350

[124] Brigati C , Giacca M , Noonan DM , Albini A . HIV Tat, its TARgets and the control of viral gene expression. FEMS Microbiol Lett. 2003;220(1):57‐65. 12644228 10.1016/S0378-1097(03)00067-3

[125] Clark E , Nava B , Caputi M . Tat is a multifunctional viral protein that modulates cellular gene expression and functions. Oncotarget. 2017;8(16):27569‐27581. 28187438 10.18632/oncotarget.15174 PMC5432358

[126] Marino J , Maubert ME , Mele AR , Spector C , Wigdahl B , Nonnemacher MR . Functional impact of HIV‐1 Tat on cells of the CNS and its role in HAND. Cell Mol Life Sci. 2020;77(24):5079‐5099. 32577796 10.1007/s00018-020-03561-4 PMC7674201

[127] Ensoli B , Moretti S , Borsetti A , et al. New insights into pathogenesis point to HIV‐1 Tat as a key vaccine target. Arch Virol. 2021;166(11):2955‐2974. 34390393 10.1007/s00705-021-05158-z PMC8363864

[128] Cafaro A , Tripiciano A , Picconi O , et al. Anti‐Tat immunity in HIV‐1 infection: effects of naturally occurring and vaccine‐induced antibodies against tat on the course of the disease. Vaccines. 2019;7(3):99. 31454973 10.3390/vaccines7030099 PMC6789840

[129] Letvin NL . Strategies for an HIV vaccine. J Clin Invest. 2002;110(1):15‐27. 12093882 10.1172/JCI15985 PMC151036

[130] Kaseke C , Tano‐Menka R , Senjobe F , Gaiha GD . The emerging role for CTL epitope specificity in HIV cure efforts. J Infect Dis. 2021;223(suppl 1):S32‐S37. 10.1093/infdis/jiaa333 PMC7883022 33586771

[131] Garcia‐Bates TM , Palma ML , Anderko RR , et al. Dendritic cells focus CTL responses toward highly conserved and topologically important HIV‐1 epitopes. EBioMedicine. 2021;63:103175. 33450518 10.1016/j.ebiom.2020.103175 PMC7811131

[132] Li F , Finnefrock AC , Dubey SA , et al. Mapping HIV‐1 vaccine induced T‐cell responses: bias towards less‐conserved regions and potential impact on vaccine efficacy in the step study. PLoS One. 2011;6(6):e20479. 21695251 10.1371/journal.pone.0020479 PMC3112144

[133] Liu Y , McNevin J , Rolland M , et al. Conserved HIV‐1 epitopes continuously elicit subdominant cytotoxic T‐lymphocyte responses. J Infect Dis. 2009;200(12):1825‐1833. 19909083 10.1086/648401 PMC2783836

[134] Picker LJ , Lifson JD , Gale M , Hansen SG , Früh K . Programming cytomegalovirus as an HIV vaccine. Trends Immunol. 2023;44(4):287‐304. 36894436 10.1016/j.it.2023.02.001 PMC10089689

[135] Zhang P , Narayanan E , Liu Q , et al. A multiclade env–gag VLP mRNA vaccine elicits tier‐2 HIV‐1‐neutralizing antibodies and reduces the risk of heterologous SHIV infection in macaques. Nat Med. 2021;27(12):2234‐2245. 34887575 10.1038/s41591-021-01574-5

[136] Xu Y , Ferguson T , Masuda K , et al. Short carbon nanotube‐based delivery of mRNA for HIV‐1 vaccines. Biomolecules. 2023;13(7):1088. 37509124 10.3390/biom13071088 PMC10377108

[137] Mandal S , Ghosh JS , Lohani SC , et al. A long‐term stable cold‐chain‐friendly HIV mRNA vaccine encoding multi‐epitope viral protease cleavage site immunogens inducing immunogen‐specific protective T cell immunity. Emerg Microbes Infect. 2024;13(1):2377606. 38979723 10.1080/22221751.2024.2377606 PMC11259082

[138] Denton PW , Garcia JV . Novel humanized murine models for HIV research. Curr HIV/AIDS Rep. 2009;6(1):13‐19. 19149992 10.1007/s11904-009-0003-2 PMC3739285

[139] Hatziioannou T , Evans DT . Animal models for HIV/AIDS research. Nat Rev Microbiol. 2012;10(12):852‐867. 23154262 10.1038/nrmicro2911 PMC4334372

[140] Sui Y , Gordon S , Franchini G , Berzofsky JA . Non‐human primate models for HIV/AIDS vaccine development. Curr Protoc Immunol Ed John E Coligan Al. 2013;102:12.14.1‐12.14.30. 10.1002/0471142735.im1214s102 PMC3920465 24510515

[141] Hirsch VM , Goldstein S , Hynes NA , et al. Prolonged clinical latency and survival of macaques given a whole inactivated simian immunodeficiency virus vaccine. J Infect Dis. 1994;170(1):51‐59. 8014520 10.1093/infdis/170.1.51

[142] Nath BM , Schumann KE , Boyer JD . The chimpanzee and other non‐human‐primate models in HIV‐1 vaccine research. TIM. 2000;8(9):426‐431. 10.1016/s0966-842x(00)01816-3 10989311

[143] Krakoff E , Gagne RB , VandeWoude S , Carver S . Variation in intra‐individual lentiviral evolution rates: a systematic review of human, nonhuman primate, and felid species. J Virol. 2019;93(16):e00538‐19. 31167917 10.1128/JVI.00538-19 PMC6675878

[144] Wertheim JO , Worobey M . Dating the age of the SIV lineages that gave rise to HIV‐1 and HIV‐2. PLoS Comput Biol. 2009;5(5):e1000377. 19412344 10.1371/journal.pcbi.1000377 PMC2669881

[145] Bell SM , Bedford T . Modern‐day SIV viral diversity generated by extensive recombination and cross‐species transmission. PLoS Pathog. 2017;13(7):e1006466. 28672035 10.1371/journal.ppat.1006466 PMC5510905

[146] Policicchio BB , Pandrea I , Apetrei C . Animal models for HIV cure research. Front Immunol. 2016;7:12. 26858716 10.3389/fimmu.2016.00012 PMC4729870

[147] Gillgrass A , Wessels JM , Yang JX , Kaushic C . Advances in humanized mouse models to improve understanding of HIV‐1 pathogenesis and immune responses. Front Immunol. 2020;11:617516. 33746940 10.3389/fimmu.2020.617516 PMC7973037

[148] Abeynaike S , Paust S . Humanized mice for the evaluation of novel HIV‐1 therapies. Front Immunol. 2021;12:636775. 33868262 10.3389/fimmu.2021.636775 PMC8047330

[149] Guenter D , Esparza J , Macklin R . Ethical considerations in international HIV vaccine trials: summary of a consultative process conducted by the Joint United Nations Programme on HIV/AIDS (UNAIDS). J Med Ethics. 2000;26(1):37‐43. 10701170 10.1136/jme.26.1.37 PMC1733173

[150] New guidance on ethical HIV prevention trials published. 2021. Accessed December 27, 2023. 2021. Accessed December 27, 2023. https://www.who.int/news/item/27-01-2021-new-guidance-on-ethical-hiv-prevention-trials-published

[151] The Development of HIV Vaccines. December 27, 2023. https://historyofvaccines.org/vaccines-101/future-immunization/development-hiv-vaccines

[152] Verywell Health. 2022. Accessed December 27, 2023. Why is it so hard to make an hiv vaccine? 2022. Accessed December 27, 2023. Why is it so hard to make an hiv vaccine? https://www.verywellhealth.com/hiv-vaccine-development-4057071

